# Newcastle Disease Virus Nonstructural V Protein Upregulates SOCS3 Expression to Facilitate Viral Replication Depending on the MEK/ERK Pathway

**DOI:** 10.3389/fcimb.2019.00317

**Published:** 2019-09-10

**Authors:** Xiangwei Wang, Yanqing Jia, Juan Ren, Na Huo, Haijin Liu, Sa Xiao, Xinglong Wang, Zengqi Yang

**Affiliations:** College of Veterinary Medicine, Northwest A&F University, Yangling, China

**Keywords:** Newcastle disease virus, SOCS3, V protein, MEK/ERK pathway, viral replication

## Abstract

Newcastle disease virus (NDV) causes serious economic losses to the poultry industry. In our previous study, we found that NDV induced a strong innate immune response in the chicken embryo and bursa of Fabricius (BF). However, the underlying mechanisms by which NDV escapes the host innate immunity are not well-understood. The suppressor of cytokine signaling 3 (SOCS3) inhibits the type I interferon-dependent antiviral signaling pathway by utilizing a feedback loop. In this study, we analyzed the transcriptome data of the chicken embryo and BF infected with NDV and found significant upregulation of SOCS3. Next, we demonstrated that NDV infection and nonstructural V protein induced the up-regulation of SOCS3. Furthermore, we showed that overexpression of SOCS3 facilitated viral replication and reduced the expression of phosphorylation STAT1, MX1, and OASL, while inhibition of SOCS3 with siRNAs reduced virus replication and promoted the expression of phosphorylation STAT1, MX1, and OASL. Finally, we demonstrated that the MEK/ERK signaling pathway was involved in the expression of SOCS3 mediated by NDV infection and V protein transfection, and using specific inhibitor U0126 to block this signaling pathway attenuated SOCS3 expression and inhibited NDV replication through promoting the expression of type I interferon, OASL and MX1. Taken together, these data demonstrate that NDV infection and NDV nonstructural V protein activates the expression of SOCS3 at the mRNA and protein level through a mechanism dependent on the MEK/ERK signaling pathway, which benefits virus replication.

## Introduction

Newcastle disease virus (NDV), non-enveloped, single-stranded negative-sense RNA virus, belongs to the *Avulavirus* genus of the *Paramyxoviridae* family (Amarasinghe et al., 2018). Although genetically diverse, all NDV strains belong to one single serotype which can be further divided into class I and class II. Since its discovery in 1926, it has caused four major epidemics around the world, and the virus remains a major threat to the worldwide poultry industry (Seal et al., 2000). Mass vaccination has been used to prevent the disease since the 1980s in China, while wild NDV is frequently discovered in commercial chickens that were immunized with NDV vaccines (Umali et al., 2013; Zhang et al., 2014; Zhu et al., 2016). Therefore, through exploring the interactions between NDV and host *in vivo* and *in vitro*, fully expounding the underlying mechanisms may provide new insights into the control of the NDV epidemic.

Activation of the innate immune system relied on the recognition of pathogen-associated molecular patterns (PAMPs) of invading viruses by specific pattern-recognition receptors (PRRs) and ultimately stimulated the host to an antiviral state (Yoneyama et al., 2004). It has been reported that intense innate immune response was induced by virulent NDV infection in chickens, including producing a mass of inflammatory factors and interferon stimulates genes (ISGs) (Hu et al., 2015; Jia et al., 2018). However, this strong host antiviral response did not clear the virus and eventually caused highly mortality in chickens. The virus completely depends on the host cells to complete its life cycle. In this process, the virus may exploit or interrupt some of the host antiviral mechanisms or host genes to escape the host immune system. The mechanism by which viruses disrupt and exploit host genes in host cells may be the key to understand the virus-host interactions.

SOCS3 belongs to suppressor of cytokine signaling (SOCS) proteins, which were shown to exert negative feedback regulation function on the JAK/STAT signaling pathway (Baker et al., 2009; Qin et al., 2016). A variety of viruses escape the host immune system by inducing the SOCS protein expression. Some previous studies reported that influenza A virus upregulated SOCS1 and SOCS3 expression and inhibited STAT 1-3 signaling by NS1 protein to promote virus replication (Jia et al., 2010). Additionally, phosphorylation of STAT1 was promoted in SOCS3 knockdown cells, and the expression of interferon-stimulated genes (ISGs) was also increased (Pauli et al., 2008). However, the potential molecular mechanism by which NDV activates SOCS3 expression is unclear.

Thus, in this study, we focused on analyzing the expression of SOCS3 upon NDV infection and the effects of SOCS3 on NDV replication. Next, we explored the possible involvement of NDV viral proteins in SOCS3 upregulation induced by NDV infection. Our results showed that NDV V protein promoted the expression of SOCS3 and overexpression of SOCS3 promoted viral replication via downregulating the expression of OASL and MX1. Finally, we demonstrated that the MEK/ERK signaling pathway played a critical role in NDV-induced SOCS3 expression. Taken together, our study provides insight into the novel role of V protein in escaping the host innate immune system during NDV infection.

## Materials and Methods

### Ethics Statement

Ten-day-old specific pathogen-free (SPF) chicken embryos (JINAN SAIS POULTRY CO., LTD.) were used to make primary chicken embryonic fibroblast cells (CEFs). All chicken embryos were handled according to the Ethics Committee at Northwest A&F University. Experiments were carried out in accordance with the approved guidelines.

### Virus

The virulent NDV F48E9, belonging to genotype IX of class II, used in this study was conserved in our laboratory and propagated in the 10-day-old specific pathogen-free (SPF) chicken embryos. The virus was stored at −80°C until further use.

### Vector Construction and Cell Transfection

The plasmids expressing NDV viral structural proteins (NP, P, M, F, HN) and non-structural protein (V) were constructed into the pCAGGS-Flag vector in our laboratory. The full-length SOCS3 gene (NCBI accession No.: NM_204600) was amplified from CEF cells using high-fidelity enzyme PrimeSTAR Max (TaKaRa, DL, China) and inserted into pCMV-HA (EcoRI, BglII) to generate pCMV-HA-SOCS3. The siRNA sequences targeting SOCS3 were designed and synthesized with the help of the Sangon Company (Shanghai, China). The primer sequences used in this study are listed in Table 1. For the transfection assay, 5 × 10^5^ CEF cells/well were cultured in a 12-well plate overnight and then transfected with 1.5 μg plasmids or 20 pmol siRNAs using 3 μl Lipofectamine 2000 (Invitrogen, Carlsbad, CA, USA) according to the instruction. Twenty-four hours after transfection, transfected cells were infected with 0.1 MOI of NDV. Supernatant and cells were collected 24 h post infection and stored for further analysis.

**Table 1:** Primers and siRNA sequence information.

**Name**	**Forward primer (5^**′**^-3^**′**^)**	**Reverse primers (5^**′**^-3^**′**^)**
SOCS3 (clone)	CCGGAATTC ATGGTCACCCACAGCAAGTTC	GGTAGATCT TTAGAGGGGGGCATCGTACTG
IFN-α (qRT-PCR)	GACATGGCTCCCACACTACC	AGGCGCTGTAATCGTTGTCT
IFN-β (qRT-PCR)	GCTCACCTCAGCATCAACAA	GGGTGTTGAGACGTTTGGAT
OASL (qRT-PCR)	AGATGTTGAAGCCGAAGTACCC	CTGAAGTCCTCCCTGCCTGT
MX1 (qRT-PCR)	AAGCCTGAGCATGAGCAGAA	TCTCAGGCTGTCAACAAGATCAA
NC	UUCUCCGAACGUGUCACGUTT	ACGUGACACGUUCGGAGA ATT
siSOCS3 #1 (218)	GCUCUAAGAGCGAGUACCATT	UGGUACUCGCUCUUAGAGCTT
siSOCS3 #2 (576)	CAAACGCACCUACUACAUUTT	AAUGUAGUAGGUGCGUUUGTT
siSOCS3 #3(697)	GACUCCUACGAGAAGAUGATT	UCAUCUUCUCGUAGGAGUCTT

### Quantitative Real-Time PCR (qRT-PCR) Analysis

Quantitative real-time PCR was carried out with a real-time thermocycler (Four-channel, Tianlong, China) using the RealStar Green Fast Mixture (GenStar, Beijing, China) according to the manufacturer's instructions. Relative expression of the target mRNAs was calculated using the 2–ΔΔCT method as previously described (Livak and Schmittgen, 2001), and the 28S gene was used to normalize the fold changes in expression.

### Tissue Culture Infection Doses (TCID50)

Viral titers were measured by TCID50. In brief, CEF cells attained a density of 85% in 96-well plates and were incubated with the cell supernatants of different groups, which were 10-fold serial dilutions, after NDV infection for 1 h, and each dilution had five replicates. The supernatants were replaced with DMEM containing 2% fetal bovine serum (FBS). After 3–5 days, the result was calculated according to the Reed-Muench method (Pizzi, 1950).

### Western Blot Analysis

Cells from different groups were lysed with 1 × SDS sample buffer, boiled for 10 min and separated by 12% SDS-PAGE, and were then transferred to a nitrocellulose membrane (Millipore, Billerica, MA, USA). After blocking for one night at 4°C with 10% skim milk, the membranes were incubated with diluted primary and secondary antibodies for 2 and 1 h at room temperature, respectively. The result was detected with Clarity Western ECL Substrate (Bio-Rad, Hercules, CA, USA). The antibodies were as follows: Mouse anti HA-tag (1:1000; Invitrogen, Carlsbad, CA, USA), mouse anti-SOCS3 (1:500; polyclonal antibody prepared in our laboratory), mouse anti-HN (1:1000; monoclonal antibody prepared in our laboratory), rabbit anti-phospho-STAT1 (1:500; ZEN BIO, Chengdu, China), mouse anti-STAT1 (1:500; ZEN BIO, Chengdu, China), rabbit anti-ERK1/2 (1:500; ZEN BIO, Chengdu, China), rabbit anti-phospho-ERK1/2 (1:500; ZEN BIO, Chengdu, China), mouse anti-β-Tubulin (1:2000; Sungene Biotech, Tianjin, China) and goat anti-mouse (1:5000; Sungene Biotech, Tianjin, China).

### Immunofluorescence Assay (IFA)

Briefly, CEF cells were fixed with 4% formaldehyde dissolved in phosphate-buffered saline (PBS) for 15 min and permeabilized with 0.5% Triton X-100 for 15 min at room temperature. After that, cells were blocked with 1% bovine serum albumin for 2 h at 37°C. Then, the cells were incubated with the primary antibodies of mouse anti-SOCS3 (1:500) and rabbit anti-NP (1:200) for one night at 4°C. The cells were incubated with DAPI after incubation with Goat anti-Mouse IgG H&L Alexa Fluor 594 (Abcam, Cambridge, UK) and Goat anti-Rabbit H&L Alexa Fluor 488 (Abcam, Cambridge, UK) for 1 h at 37°C. The fluorescence was observed in a fluorescence microscope.

### Cell Viability Assay

CCK-8 kit (BestBio, China) was used to detect the cell viability (Lv et al., 2018). In brief, CEF cells in 96-well plates were treated with 20 μM U0126 dissolved in DMSO for 24 and 48 h. Then, 10 μl CCK-8 reagent was added into the cells and incubated in the dark for 2 h at 37°C. A spectrophotometer (Bio-Rad) was used to read the OD_450_ of each well. Viabilities of the CEF cells treated with U0126 were compared with that of negative control.

### Statistical Analysis

All data presented were from at least three independent experiments. Student's *t*-test was performed for statistical analysis using the GraphPad Prism 5 software (San Diego, CA, USA). The statistical differences are presented as follows: ^*^P < 0.05, ^**^P < 0.01.

## Results

### NDV Infection Promotes the Expression of SOCS3 in CEF Cells

**Figure 1:**
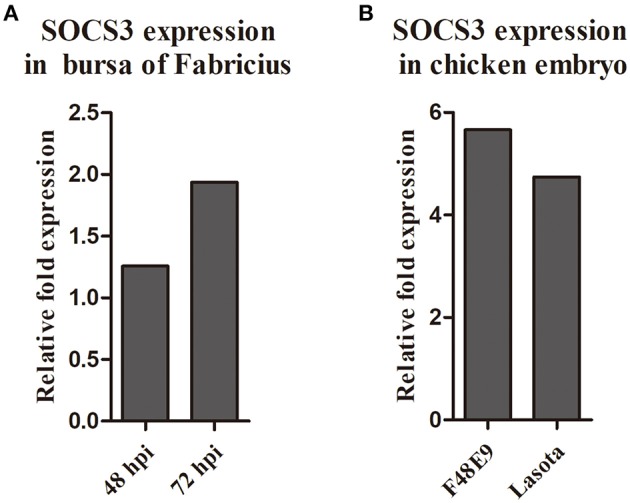
The expression of SOCS3 in the transcriptome sequencing of the chick embryo **(A)** and bursa of Fabricius **(B)**.

**Figure 2:**
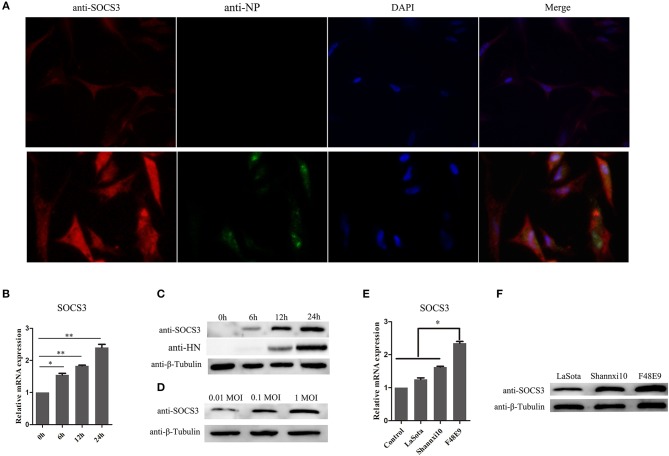
NDV infection increases the expression of SOCS3. **(A)** CEF cells were infected with NDV (0.1 MOI). IFA was performed to detect the expression of SOCS3 and NDV 24 hpi. **(B,C)** CEF cells were infected with NDV (0.1 MOI), cells were harvested and qRT-PCR and WB were performed to test the expression of SOCS3 at different times. **(D)** WB was performed to detect the protein expression of SOCS3 at 24 h post different MOI NDV (0.01 MOI, 0.1 MOI, 1 MOI) infection. **(E,F)** CEF cells were infected with different virulence NDV. At 24 hpi, cells were harvested and qRT-PCR and WB were performed to test the expression of SOCS3. Data presented are from three independent experiments and presented as mean ± SD. *P < 0.05; **P < 0.01.

We analyzed our transcriptome sequencing data of the chick embryo (Jia et al., 2018) and bursa of Fabricius (unpublished data) and found that SOCS3 was significantly upregulated (Figure 1). To verify whether SOCS3 expression can be activated by NDV infection, NDV-infected cells were subjected to immunofluorescence assay. An increased expression of SOCS3 (red fluorescence) could be seen in NDV-infected cells (green fluorescence) (Figure 2A). We next analyzed the mRNA expression of SOCS3 at different time points after NDV infection in CEF cells. As shown in Figures 2B,C, an obvious increase of SOCS3 mRNA and protein expression could be detected in a time-dependent manner from 6 h after NDV infection. Meanwhile, the expression of SOCS3 is dose-dependent with virus titer (Figure 2D). To detect whether different virulence NDV strains affected the level of SOCS3 expression, CEF cells were infected with 0.1 MOI of LaSota (lentogenic stain), Shannxi10 (mesogenic stain), and F48E9 (velogenic stain), and cell lysates were collected 24 hpi for further analysis. As shown in Figures 2E,F, there were significant differences in inducing the production of SOCS3 among the strains with different virulence, and F48E9 induced SOCS3 expression is higher than the others. These data suggested that NDV infection upregulated SOCS3 expression.

### NDV V Protein Upregulates the Expression of SOCS3

**Figure 3:**
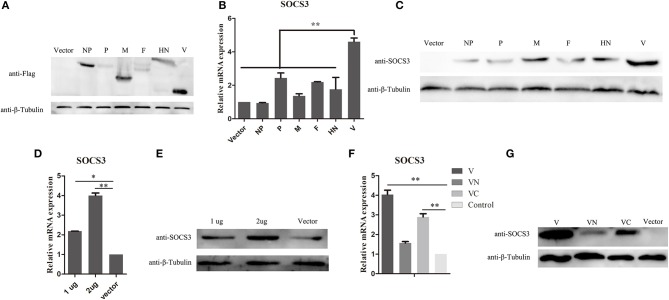
NDV V protein plays a vital role in upregulating the expression of SOCS3. **(A–C)** CEF cells were transfected with plasmids expressing FLAG-tagged NDV genes. Cells were harvested and qRT-PCR and WB were performed to detect the expression of SOCS3 24 h post transfection. **(D,E)** CEF cells were transfected with pCAGGS-Flag (Vector) and pCAGGS-Flag-V (1, 2 μg). Cells were harvested and qRT-PCR and WB were performed to detect the expression of SOCS3 24 h post transfection. **(F,G)** CEF cells were transfected with pCAGGS-Flag (Vector) and pCAGGS-Flag-V (V), pCAGGS-Flag-VN (VN), and pCAGGS-Flag-VC (VC). Cells were harvested and qRT-PCR and WB were performed to detect the expression of SOCS3 24 h post transfection. Data presented are from three independent experiments and presented as mean ± SD. *P < 0.05; **P < 0.01.

NDV infection can induce the expression of SOCS3 at the mRNA and protein levels. To further identify which component of NDV was involved in the upregulation of SOCS3 in CEF cells, the eukaryotic expression vector providing structural proteins and non-structural protein was constructed and transfected into CEF cells to identify their roles in inducing SOCS3 expression (Figure 3A). As shown in Figure 3B, we did not detect notable changes of SOCS3 mRNA expression in these transfected cells, except for the V protein. The V protein could markedly increase the expression of SOCS3 in mRNA level. Next, we transfected CEF cells with viral protein-expressing plasmids and vector control and confirmed the expression of SOCS3 at the protein level. As shown in Figure 3C, we detected that V protein transfected cells induced notable changes in the expression of SOCS3 in contrast to other viral protein transfected cells, demonstrating that the V protein played critical roles in inducing the expression of SOCS3 during NDV infection. Further, CEF cells were then transfected with different amounts of plasmid-expressed V protein to detect the expression of SOCS3. We found that the plasmid-expressed V protein upregulated expression of SOCS3 in mRNA and protein level by a dose-dependent manner (Figures 3D,E), and the C terminal of V protein plays a significant role in inducing the expression of SOCS3 (Figures 3F,G). Collectively, these data suggest that NDV V protein played a vital role in inducing the expression of SOCS3.

### Overexpression of SOCS3 Promoted NDV Replication

**Figure 4:**
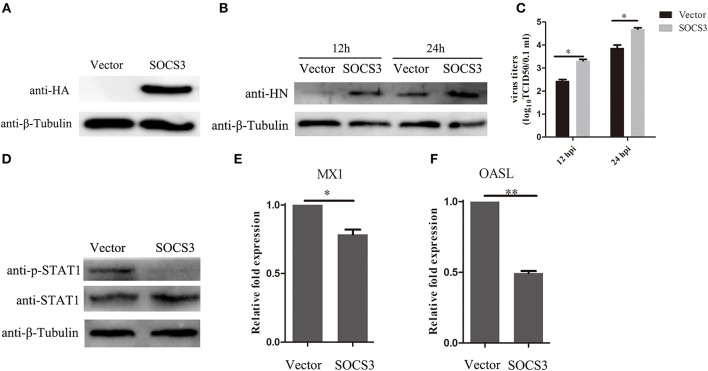
Overexpression of SOCS3 promotes NDV replication. CEF cells were transfected with pCMV-SOCS3-HA and pCMV-HA. At 24 h post transfection, CEF cells were infected NDV (0.1 MOI), cells and supernatant were harvested at 12 and 24 hpi. **(A,B)** WB was performed to detect the expression of exogenous SOCS3 and HN protein of NDV. **(C)** TCID50 was performed to measure viral RNA replication. **(D)** WB was performed to detect the expression of STAT1 and phosphorylated STAT1 in SOCS3-overexpressed cells at 24 hpi. **(E,F)** qRT-PCR was performed to detect the expression of MX1 and OASL. Data presented are from three independent experiments and presented as mean ± SD. *P < 0.5, **P < 0.01.

Since we found that NDV infection upregulated the expression of SOCS3, this prompted us to detect the function of SOCS3 during NDV infection. Then, we constructed the eukaryotic expression vector expressing SOCS3 (pCMV-HA-SOCS3) and transfected into CEF cells (Figure 4A). Next the SOCS3-overexpressed cells were infected with NDV and then virus replication was detected at 24 hpi. First, we analyzed the expression of HN protein of NDV in cells at 12 and 24 hpi and found an improvement in HN protein levels in SOCS3-overexpressed cells (Figure 4B). The virus titers were also significantly improved at 12 and 24 hpi (Figure 4C). In addition, SOCS3 has been demonstrated to inhibit the type I interferon-dependent antiviral signaling pathway by utilizing a feedback loop (Vlotides et al., 2004). Therefore, we detected the role of SOCS3 in STAT1 expression in CEF cells during NDV infection. As shown in Figure 4D, overexpression of SOCS3 could significantly reduce the phosphorylation expression of STAT1 after NDV infection. In addition, the result of qRT-PCR showed a lower level of OASL and MX1 expression in SOCS3-overexpression cells than the control group (Figures 4E,F). These data suggest that SOCS3 can promote NDV replication through inhibiting the expression of OASL and Mx1.

### Knockdown of SOCS3 Decreased NDV Replication

**Figure 5:**
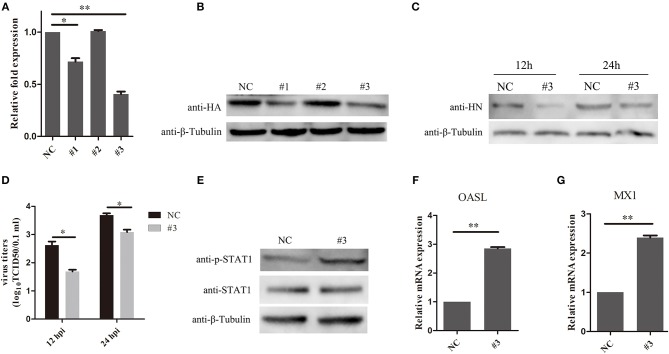
Knock-down of SOCS3 inhibits NDV replication. **(A,B)** CEF cells were co-transfected with a pCMV-SOCS3-HA expression plasmid and non-targeting control siRNA (NC) or SOCS3-targeting siRNAs, followed by qRT-PCR and WB using an anti-HA antibody to compare the efficiencies of these three siRNAs. **(C,D)** CEF cells were transfected with NC or #3. CEF cells were infected NDV (0.1 MOI) 24 h post transfection, and cells and supernatant were harvested at 12 and 24 hpi. WB was performed to detect the expression of HN protein of NDV **(C)**. TCID50 was used to measure viral RNA replication **(D)**. **(E)** WB was performed to detect the expression of STAT1 and phosphorylated STAT1 in SOCS3-knockdown cells at 24 hpi. **(F,G)** qRT-PCR was used to detect the expression of MX1 and OASL. Data presented are from three independent experiments and presented as mean ± SD. *P < 0.5, **P < 0.01.

To further examine the effects of SOCS3 on NDV replication, CEF cells were transfected with short interfering RNAs (si-SOCS3 and si-NC), which targeted SOCS3 mRNA. The results of qRT-PCR suggested that two of the si-SOCS3 could effectively decreased the endogenous expression of the SOCS3 mRNA (Figure 5A). Next we co-transfected CEF cells with pCMV-HA-SOCS3 and si-SOCS3 to detect the inhibition efficiency, and the western blotting results suggested that these two si-SOCS3 also reduced the exogenous expression of HA-SOCS3 protein (Figure 5B). Next, we selected the si-SOCS3 (#3) to detect the function on NDV replication in CEF cells. The SOCS3-knockdown cells (#3) were infected with NDV and then virus replication was detected at 12 h and 24 hpi. First, we detected the expression of HN protein in cells and found a decrease in HN protein levels in SOCS3-knockdown cells (Figure 5C). The virus titers were also significantly decreased at 12 and 24 hpi (Figure 5D). Next, we detected the effect on STAT1 expression after NDV infection in SOCS3-knockdown cells. As shown in Figure 5E, in SOCS3-knockdown cells enhanced the phosphorylation expression of STAT1. In addition, the result of qRT-PCR showed a significantly increased level of OASL and MX1 expression in SOCS3-knockdown cells compared to the control group (Figures 5F,G). These results clearly establish the role of SOCS3 in promoting NDV replication in cell response to NDV infection.

### MEK/ERK Pathway Was Involved in SOCS3 Expression During NDV Infection

**Figure 6:**
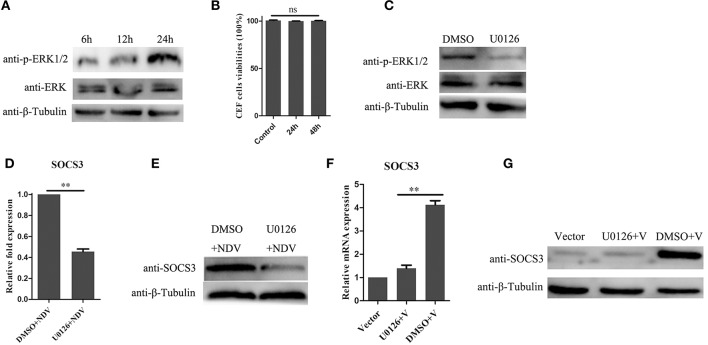
MEK/ERK signaling pathway is involved in the expression of SOCS3. **(A)** phosphorylated ERK1/2 was detected after NDV (0.1 MOI) infection at different time. **(B)** CEF cells were treated with 20 μM of the ERK 1/2 inhibitor U0126 or DMSO for 24 and 48 h, and CCK-8 assay was performed to analyze the cytotoxicity of U0126 (20 μM) to CEF cells. CEF cells were treated with 20 μM of the ERK 1/2 inhibitor U0126 or DMSO for 12 h and infected with NDV (0.1 MOI). Cells were harvested at 24 hpi. Phosphorylated ERK1/2 was detected by WB **(C)**, and qRT-PCR **(D)** and WB **(E)** were used to detect the expression of SOCS3. **(F,G)** CEF cells were treated with 20 μM of the ERK 1/2 inhibitor U0126 or DMSO for 12 h and then transfected with a pCAGGS-V-Flag (V) expression plasmid or pCAGGS-Flag (Vector). Cells were harvested 24 h post transfection. qRT-PCR **(F)** and WB **(G)** were used to detect the expression of SOCS3. Data presented are from three independent experiments and presented as mean ± SD. **P < 0.01.

In previous studies, the MEK/ERK signaling pathway was reported to be involved in expression of SOCS3 (Borland et al., 2009; Ehlting et al., 2015). In this study, we found that NDV infection could activate the MEK/ERK signaling pathway in CEF cells (Figure 6A). To determine whether the MEK/ERK signaling pathway was involved in NDV-induced expression of SOCS3, we treated CEF cells with U0126, which is a small molecule inhibitor for MEK and inhibits the phosphorylation of ERK1/2. First, we tested whether the U0126 at the concentration of 20 μM we used led to cell death, and as shown in Figures 6B,C, the U0126 had no influence on cell viability and could significantly block the phosphorylation of ERK1/2 induced by NDV infection. Next we treated the CEF cells with the U0126 for 12 h and then infected with NDV for 24 h. As shown in Figure 6D, the result of qRT-PCR showed that U0126 could inhibit the upregulation of SOCS3 mRNA during NDV infection. Furthermore, U0126 decreased the expression of SOCS3 protein (Figure 6E). Similarly, as shown in Figures 6F,G, U0126 also inhibited the expression of SOCS3 in mRNA and protein level mediated by V protein. These results indicate that the NDV induced the expression of SOCS3 depending on the MEK/ERK signaling pathway.

### Inhibition of MEK/ERK Pathway Enhanced the Expression of Antiviral Genes

**Figure 7:**
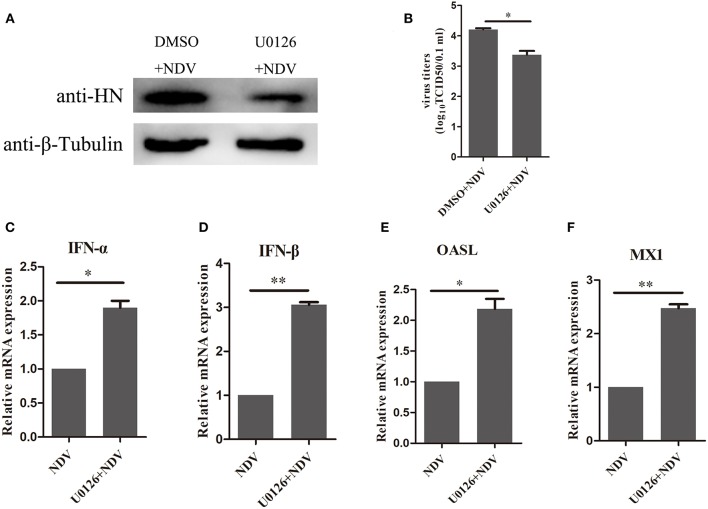
Inhibition of MEK/ERK pathway inhibited NDV replication by improving the expression of antiviral genes. CEF cells were treated with 20 μM of the ERK 1/2 inhibitor U0126 or DMSO for 12 h and infected with NDV (0.1 MOI). Cells and cellular supernatant were harvested at 24 hpi. WB **(A)** and TCID50 **(B)** was used to detect NDV replication. **(C–F)** qRT-PCR was used to detect the expression of IFNα, IFNβ, OASL, and MX1. Data presented are from three independent experiments and presented as mean ± SD. *P < 0.5, **P < 0.01.

The above result indicated that the MEK/ERK signaling pathway is involved in the expression of SOCS3 during NDV infection and that MEK inhibitor U0126 could attenuate SOCS3 expression by NDV infection and V protein transfection. Therefore, we wonder about the potential role of U0126 on NDV infection. As shown in Figure 7A, treatment with the MEK inhibitor U0126 reduced NDV HN protein expression compared with the DMSO treated cells, and the virus titers were significantly decreased in U0126-treated cells in comparison to the control group (Figure 7B). Further, we detected the expression of type I interferon and ISGs. As shown in Figures 7C–F, U0126 treatment could enhance the expression of type I interferon and ISGs. Collectively, these results demonstrated that inhibition of the MEK/ERK signaling pathway reduced the expression of SOCS3 and then enhanced the expression of the antiviral genes and further led to inhibition of the replication of NDV.

## Discussion

Interferons play important antiviral roles in host innate immune response, and diverse strategies have been evolved by NDV infection to escape the innate immune infection (Motz et al., 2013; Qiu et al., 2016). Previous studies have shown that NDV infection induces intense innate immunity response in chick embryos along with highly expressed SOCS3, which was shown to be exert negative feedback regulation function on the JAK/STAT signaling pathway (Jia et al., 2018). We suspected that the intense innate immunity response's lack of success in clearing NDV infection is related to high expression of SOCS3 protein.

SOCS family proteins play an important role in negative regulating JAK-STAT signaling pathway. Here, NDV infection upregulated expression of SOCS3, with the expression level of SOCS3 related to the virulence of NDV strains (Figure 2). Over-expression of SOCS3 inhibited antiviral gene OASL and MX1 expression, allowing efficient NDV replication. Knockdown of the expression of SOCS3 with siRNA induced the upregulation of antiviral gene OASL and MX1 expression and decreased the replication of NDV, indicating that SOCS3 plays critical roles in negatively regulating the ISGs' expression during NDV infection. Previous studies have demonstrated that many viruses induce the expression of SOCS3 to escape the IFN-mediated antiviral response, such as HIV (Sood et al., 2019), HCV (Collins et al., 2014), and IAV (Pothlichet et al., 2008). In this study, NDV also induced the upregulation of SOCS3 to antagonize IFN-mediated antiviral response.

We have demonstrated that NDV infection can enhance the expression of SOCS3, however the mechanism by which NDV induces the expression of SOCS3 was not well-elucidated. As the structural protein (except L protein) and non-structural protein (except W protein) of NDV have been well-studied, in this study, we wonder about their role in inducing the expression of SOCS3. As shown in Figure 2, V protein significantly upregulates the expression of SOCS3 mRNA and protein levels. The results suggested that SOCS3 was upregulated and activated by V protein upon NDV infection. As is known, among the Newcastle viral proteins, the V protein has been identified as the main type I IFN antagonistic factor *in vitro* (Motz et al., 2013). On the one hand, V protein suppresses the induction of type I IFN by interacting with an intracellular viral RNA sensor, melanoma differentiation-associated gene 5 (MDA5) and the transcription factors interferon regulatory factor-3 (IRF3) (Irie et al., 2012). On the other hand, V protein targets STAT1 degradation to block IFN signaling, reducing the expression of IFN-responsive genes (Mebatsion et al., 2001; Alamares et al., 2010). In this study, we demonstrated that on the basis of antagonistic interferon signaling pathway, V protein can also make the host produce genes beneficial to its replication and inhibit the host response that is harmful to its own proliferation.

In previous studies, the NF-kappaB and MEK/ERK signaling pathways have been shown to contribute to the regulation of SOCS3 expression (Pauli et al., 2008; Borland et al., 2009; Ehlting et al., 2015), and the role of the MEK/ERK signaling pathway in inducing SOCS3 expression during virus infection has rarely been reported. Activation of the MEK/ERK signaling pathway has been observed as a positive factor in promoting viral replication in cells during various viral infections (Albarnaz et al., 2014; Shi et al., 2014; Takahashi and Suzuki, 2015). In previous findings, the MEK/ERK signaling pathway activity could facilitate NDV replication, and V protein played a vital role in activating the pathway (Chu et al., 2018). We therefore doubted whether MEK/ERK was involved in the induction of SOCS3 during NDV infection. It was shown that MEK inhibitor U0126 could reduce the upregulation of SOCS3 by NDV infection and V protein. Furthermore, the inhibitor U0126 could also decrease NDV replication through upregulation of IFN-I interferon and antiviral genes OASL and MX1. These results demonstrated that the MEK/ERK signaling pathway activity was needed for SOCS3 expression during NDV infection.

In conclusion, our study uncovered a significant role of NDV nonstructural protein V in assisting the virus to escape the host innate immunity. We demonstrated that NDV infection and V protein increased the expression of SOCS3 to promote its viral replication. Furthermore, we demonstrated that the MEK/ERK signaling pathway plays an important role in the production of SOCS3 induced by NDV infection. Taken together, these data demonstrate that NDV infection and NDV nonstructural V protein activate the expression of SOCS3 at the mRNA and protein level through a mechanism depending on the MEK/ERK signaling pathway, which benefits virus replication.

## Data Availability

All datasets generated for this study are included in the manuscript.

## Ethics Statement

All embryonated chicken eggs were handled in accordance with the Ethics Committee at Northwest A&F University. Experiments were carried out in accordance with the approved guidelines.

## Author Contributions

XiaW and ZY conceived and designed the experiments. XiaW, XinW, and HL performed the experiments and drafted the manuscript. XiaW and YJ analyzed the data. JR, NH, and SX contributed to reagents and materials. All authors read and approved the final manuscript.

### Conflict of Interest Statement

The authors declare that the research was conducted in the absence of any commercial or financial relationships that could be construed as a potential conflict of interest.
